# Supplementary material for: A bionic stretchable nanogenerator for underwater sensing and energy harvesting
Source: Nat Commun. 2019 Jun 19;10:2695. doi: 10.1038/s41467-019-10433-4 (PMC6584498; doi:10.1038/s41467-019-10433-4)
Supplement: Supplementary file 1 — Supplementary Information [file 41467_2019_10433_MOESM1_ESM.pdf]

Supplementary Information

A bionic stretchable nanogenerator for underwater sensing and energy harvesting

Zou et al.

Supplementary Figures

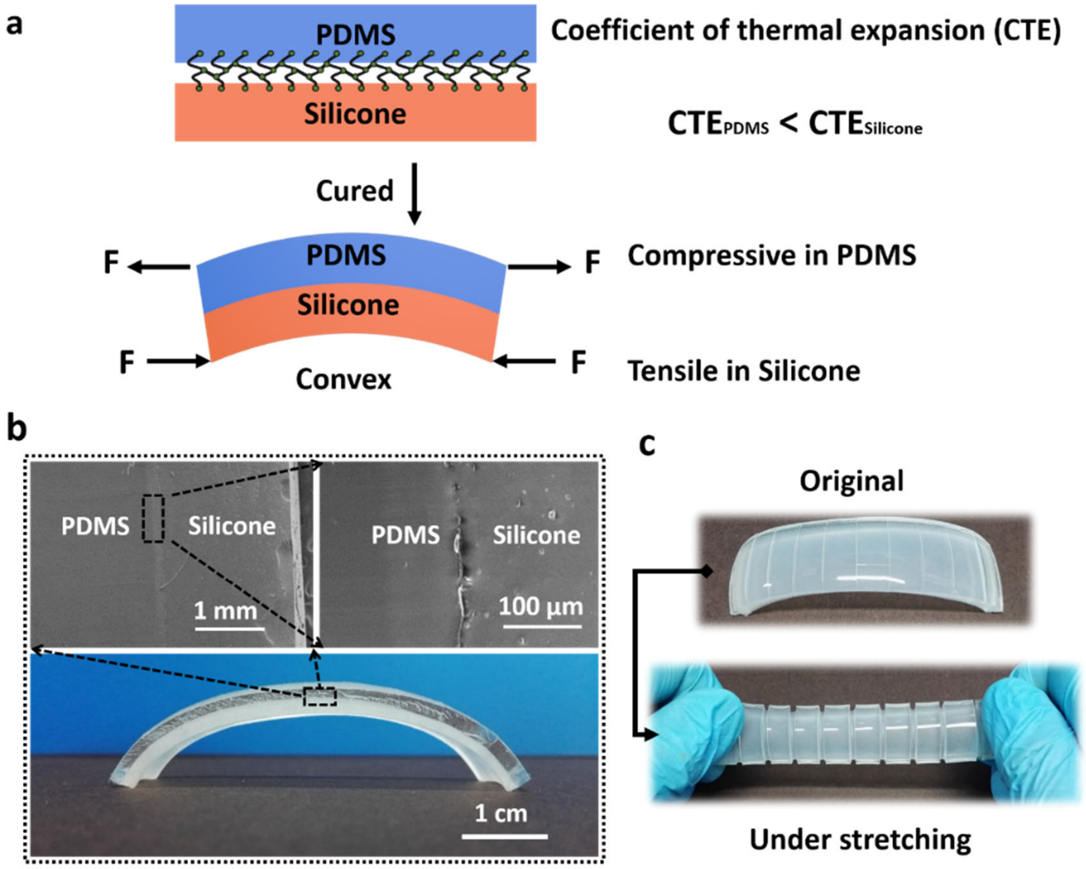

Supplementary Figure 1 Phenomenon of stress mismatch between silicone and PDMS. (a) The mechanism of stress mismatch between silicone and PDMS. (b) Optical image and SEM of PDMS-silicone double layer composite. (c) Optical image of PDMS-silicone composite with multiple sections PDMS layer in original and stretching state.

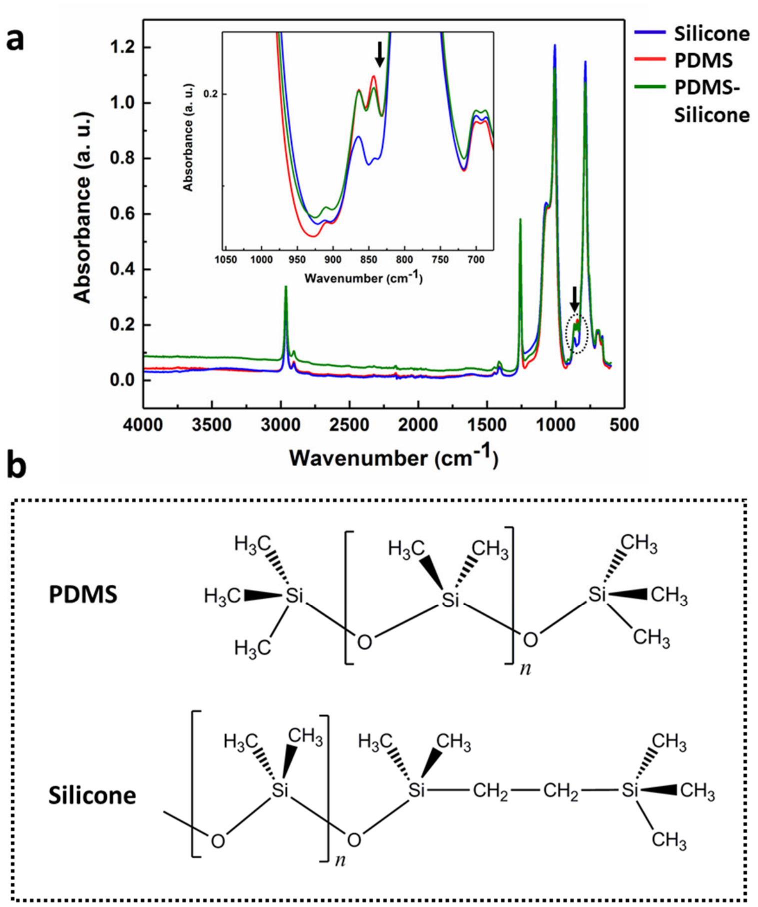

Supplementary Figure 2 Chemical characterization of the PDMS-silicone composite. (a) The infrared spectrum of PDMS, silicone and the PDMS-silicone double layer composite. (b) The structural formula of PDMS and silicone.

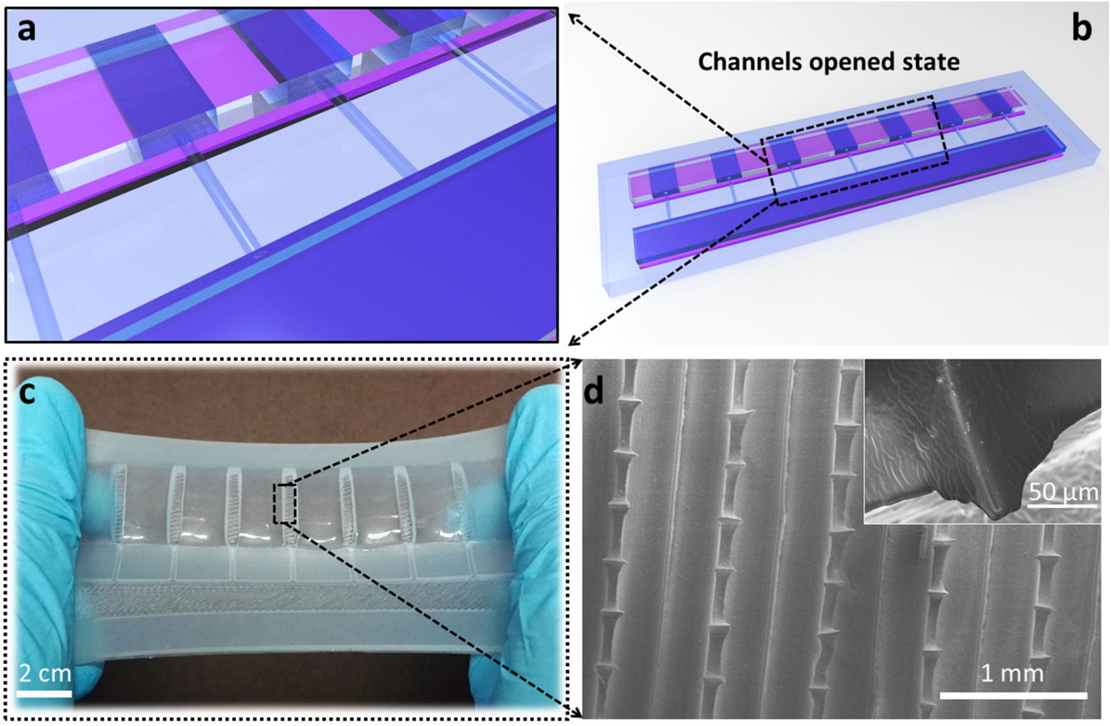

Supplementary Figure 3 Mechanosensitive multiple channels of BSNG. (a) Scheme of the mechanosensitive multiple channels of BSNG. (b) Scheme of the BSNG under stretching state. (c) Optical image of the mechanosensitive multiple channels of BSNG. (d) SEM of the micro-structure on mechanosensitive multiple channels (a high magnification image is shown in the upper right corner).

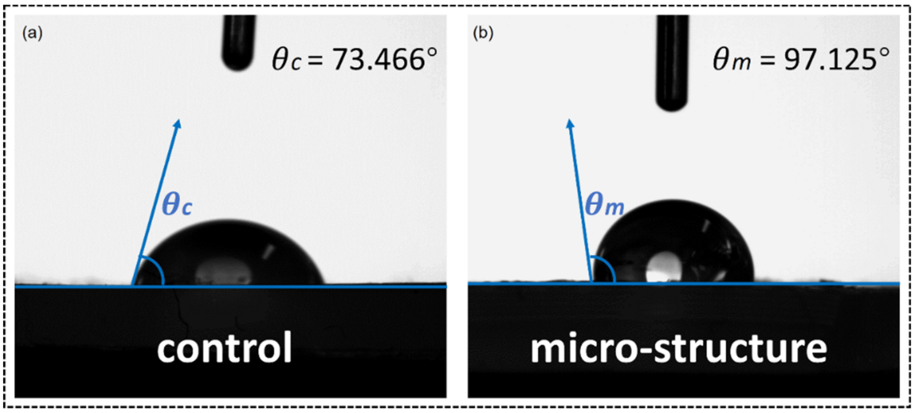

Supplementary Figure 4 Contact angle test results of (a) the silicone with smooth surface and (b) the silicone with bamboo joint-liked microstructure surface.

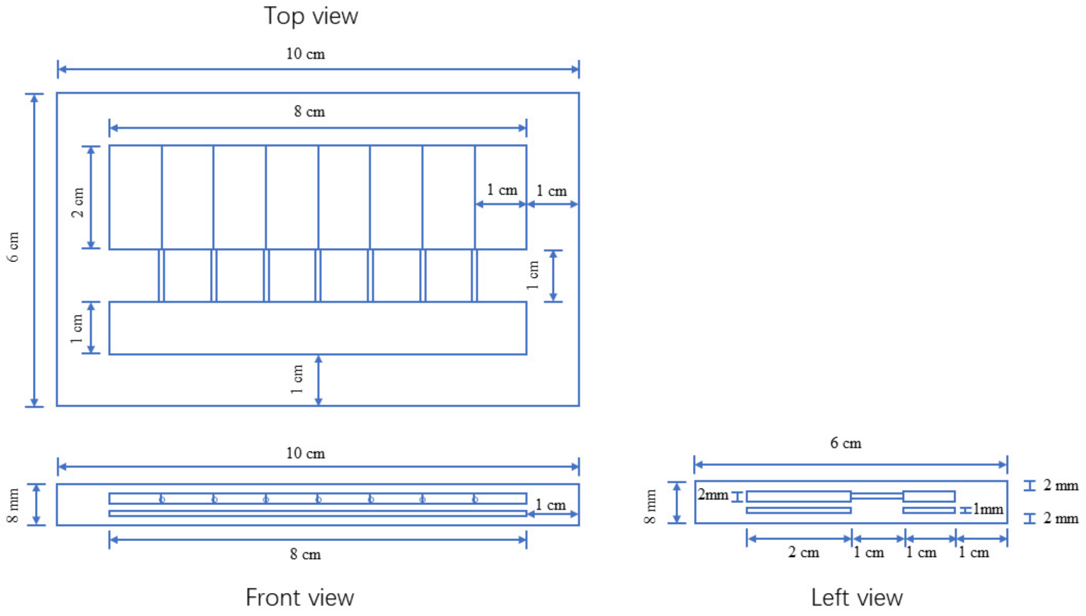

Supplementary Figure 5 Three views of BSNG and the specific dimensions of each part structure of BSNG.

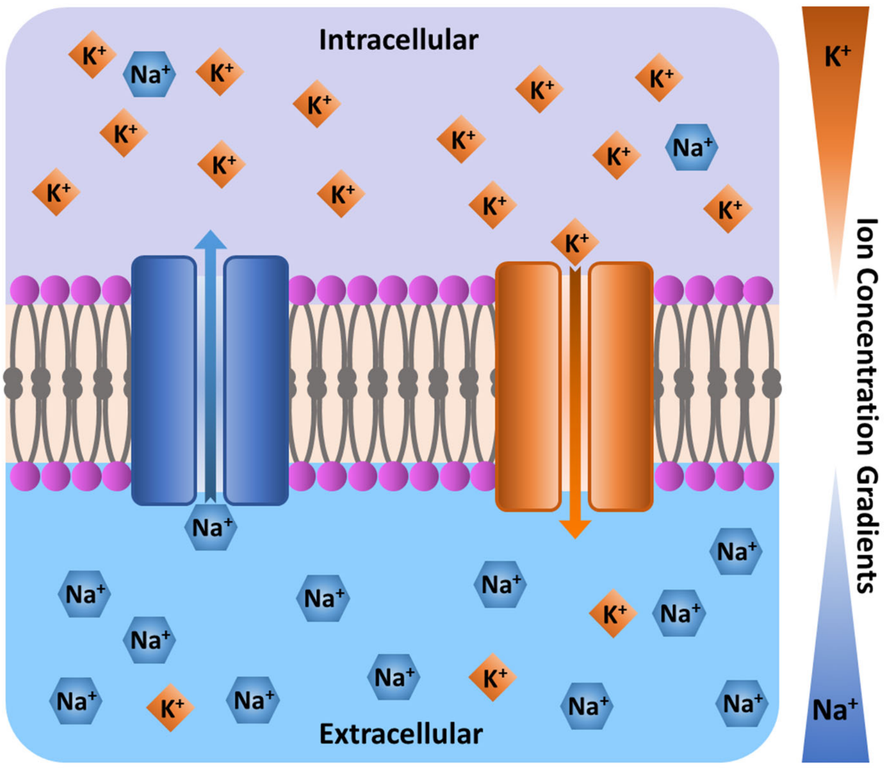

Supplementary Figure 6 Progress of Na^+ and K^+ transfer in cell membrane.

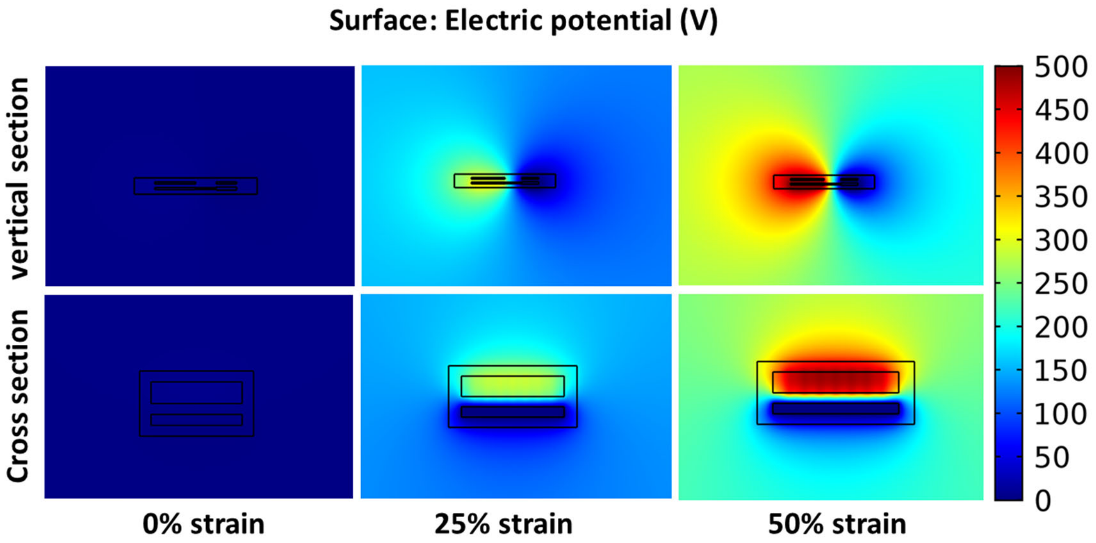

Supplementary Figure 7 Simulation result of BSNG working in liquid-solid contact mode. The changes of the surface potential of the BSNG in different strain through finite element simulation (COMSOL) are consistent with experiment results.

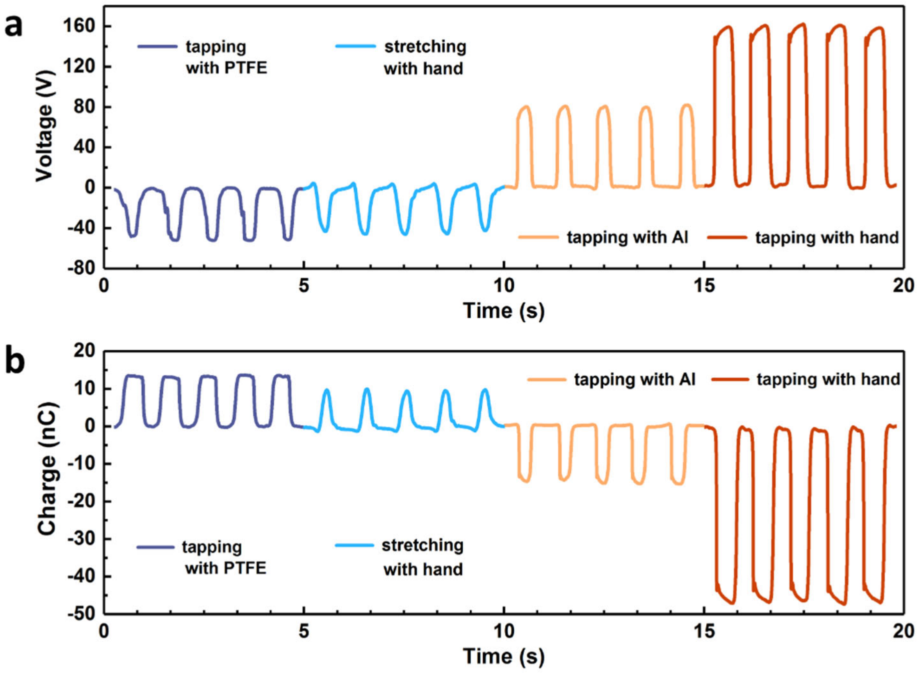

Supplementary Figure 8 Outputs of the BSNG in different working modes. (**a**) Open circuit voltage *V*oc and (**b**) short-circuit charge quantity *Q*sc of BSNG in single electrode working mode (tapping with PTFE, Al and hand) and in liquid-solid contact working mode (stretching with hand).

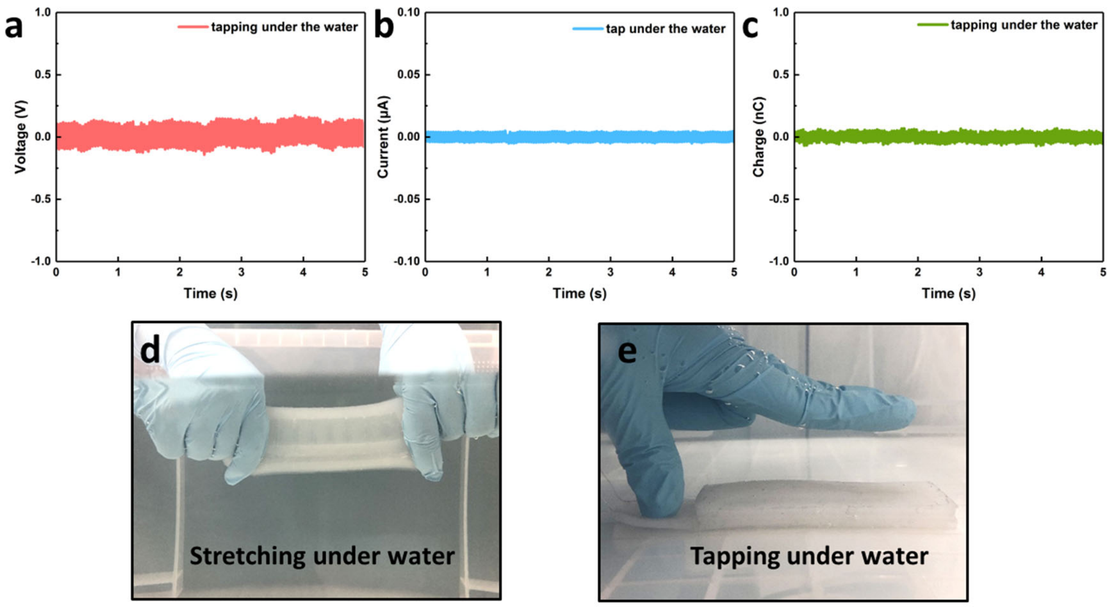

Supplementary Figure 9 Underwater output of the single-electrode mode BSNG. (**a**) Open circuit voltage *V*oc, (**b**) short-circuit current *I*sc and (**c**) short-circuit charge quantity *Q*sc of the BSNG when working in single electrode underwater. (**d**) Optical image of BSNG when stretching under water. (**e**) Optical image of BSNG when tapping under water.

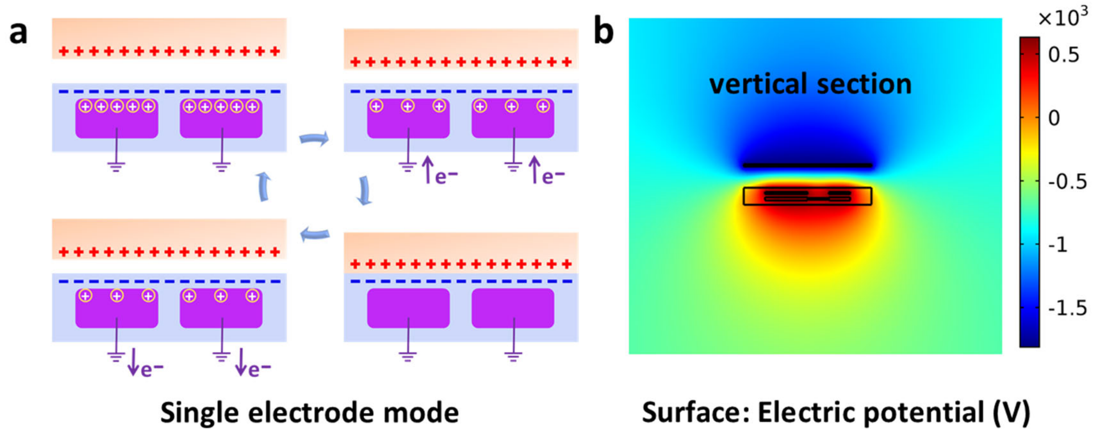

Supplementary Fig. 10. Principle of the single-electrode mode BSNG. (a) Schematic diagram of the working mechanism of SE-BSNG. (b) Simulation result of SE-BSNG through finite element simulation (COMSOL).

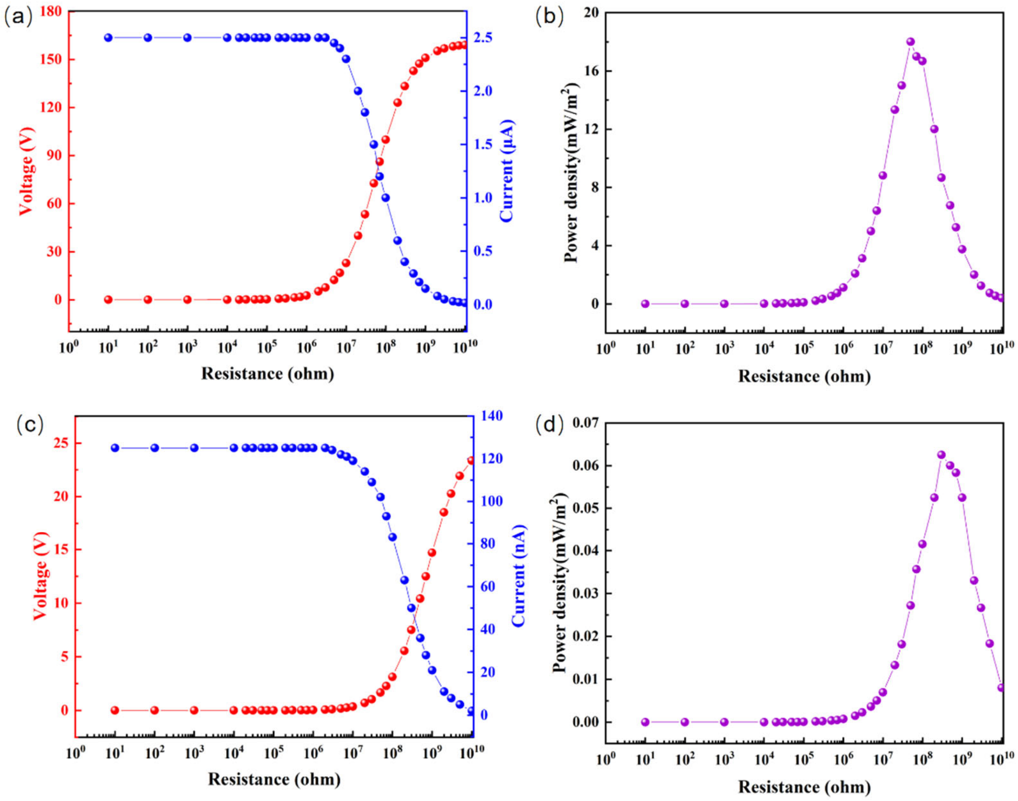

Supplementary Figure 11 Power density curves of BSNG in two different working modes. (a) Open-circuit voltage and short-circuit current for single electrode mode of BSNG at different load resistances. (b) Peak power density for single electrode mode of BSNG at different load resistances. (c) Open-circuit voltage and short-circuit current for liquid-solid contact mode of BSNG at different load resistances. (d) Peak power density for liquid-solid contact mode of BSNG at different load resistances.

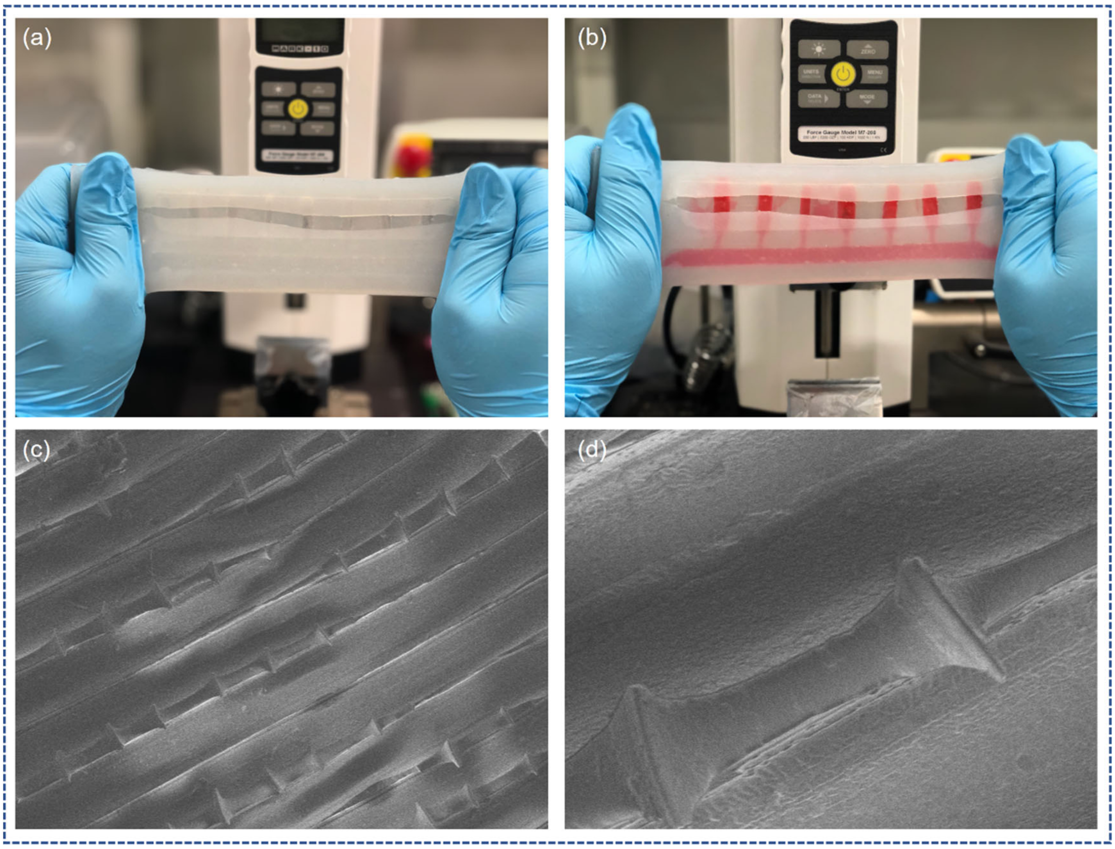

Supplementary Figure 12 Characterization of the internal structure of BSNG after 50,000 times fatigue test. (a-b) Optical photos of the internal structure of BSNG, (c-d) SEM photos of the bionic channels of BSNG.

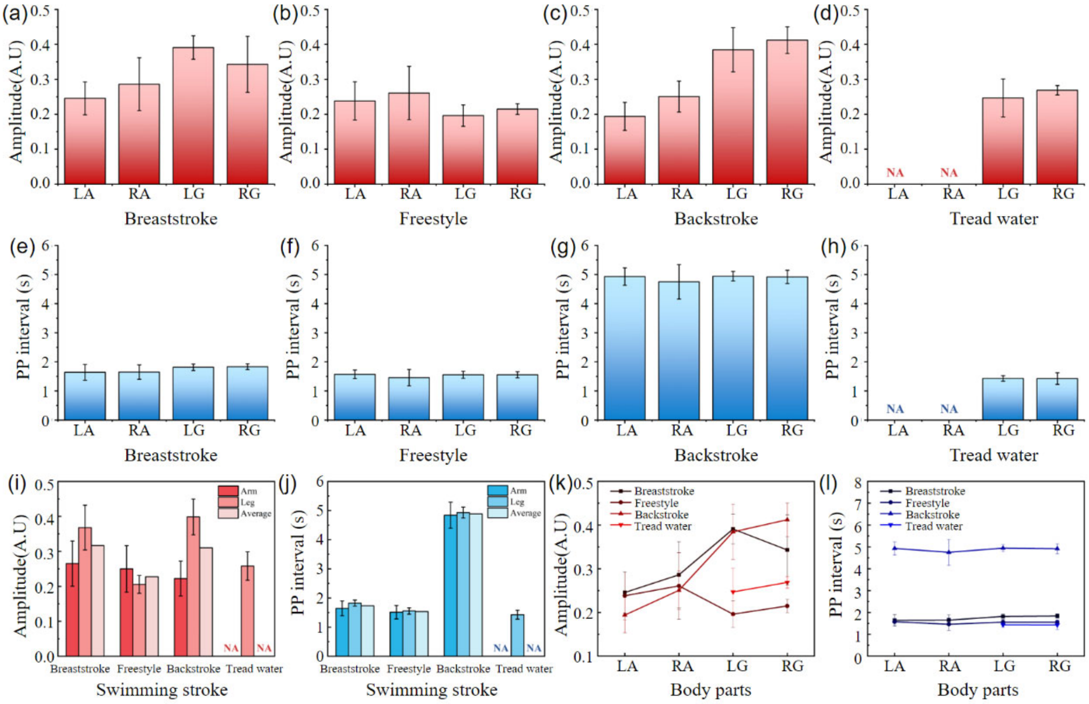

Supplementary Figure 13 Statistical analysis of motion signals in four strokes. (a-d) Average motion amplitude of the motion signals of four strokes. (e-h) Average time interval of the peaks of the motion signals of four strokes. (i) Statistical average motion amplitude of the motion signals of four strokes. (j) Statistical average time interval of the peaks of the motion signals of four strokes. (k) Average motion amplitude of different body parts in four strokes. (l) Average time interval of different body parts in four strokes. Source data of (a-l) are provided as a Source Data file. All data in (a-l) are presented as mean ± s.d.

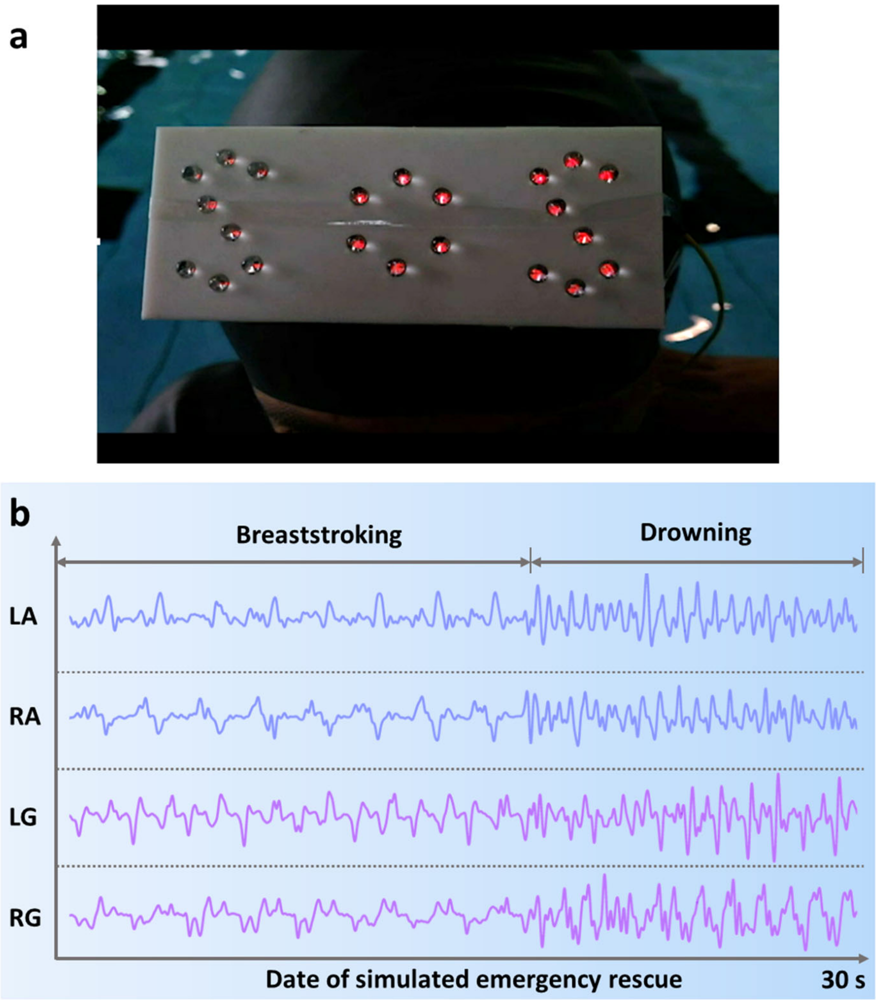

Supplementary Figure 14 Additional rescue signals and recorded information based on BSNG. (a) A SOS rescue light lighted by tapping the BSNG on the shoulder of swimmer. (b) Signals of simulated drowning process of swimmer recorded by the wireless motion monitoring system.

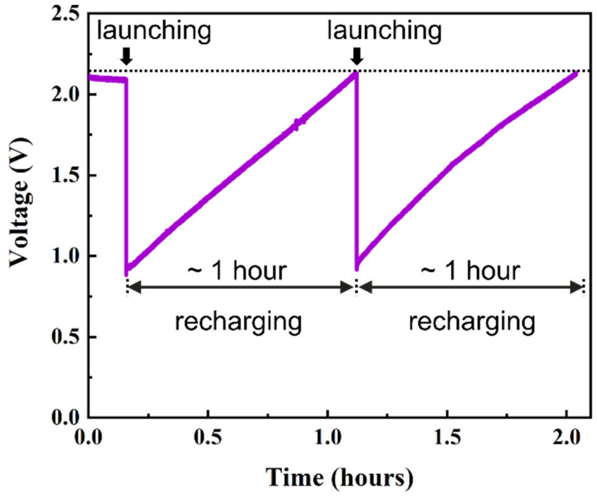

Supplementary Figure 15 Recharging a 100 μF capacitor by BSNG after the transmitter launching a wireless signal.

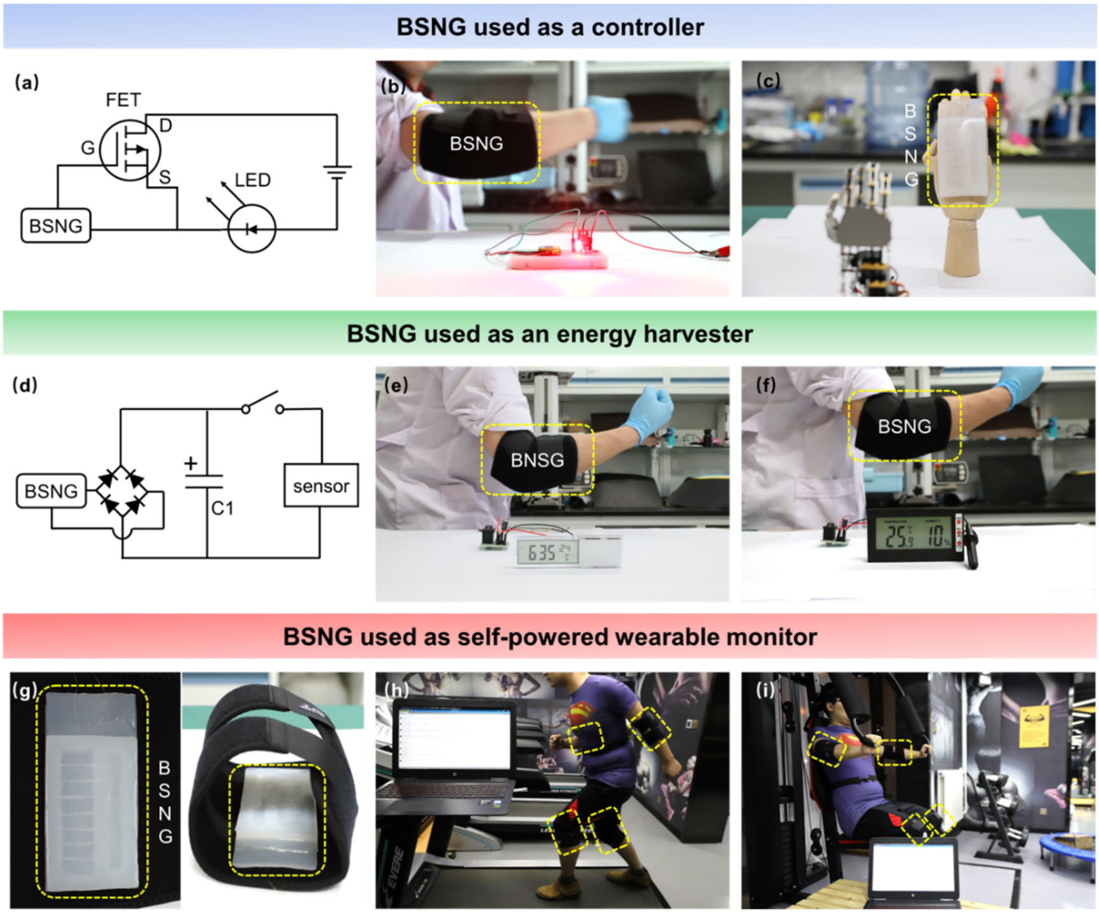

Supplementary Figure 16 Three types of applications for BSNG. (a-c) BSNG used as a controller and the outlook for BSNG used as electronic skin. (d-f) BSNG used as an energy harvester to drive some low-power appliances. (g-i) BSNG used as self-

powered wearable devices for monitoring motions in human's daily life.

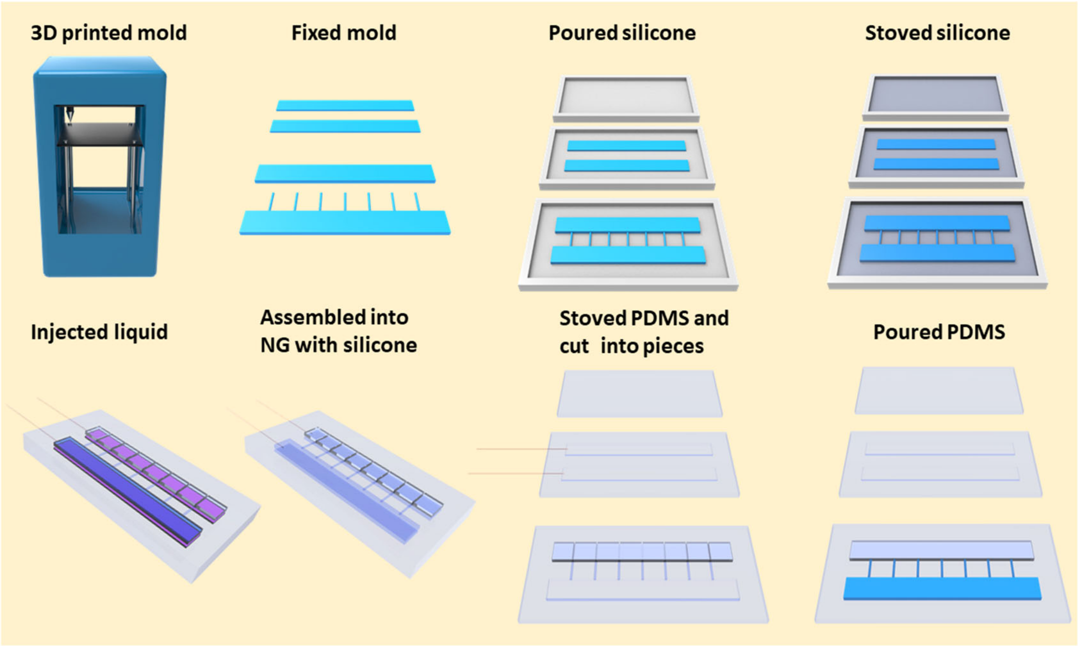

Supplementary Figure 17 Fully fabrication process of the BSNG.

Supplementary Notes

Supplementary Note 1

Phenomenon of stress mismatch between silicone and PDMS

Stress mismatch occurs when two different materials are combined together due to the differences of Young's modulus and stress-strain characteristics between each material. When the two different materials are combined together, the volume of them changes differently due to the respective characteristics of thermal expansion and contraction, leading to an interface thermal stress existing in the joint. If the two materials are combined tightly, the composite material will bend in one direction due to the mismatch thermal stress. If the bonding force of the two materials is less than the thermal stress, the interface between the two materials will be mismatched and delamination[1,2].

Thermal expansion is the tendency of matter to change its shape, area and volume, in response to a change of temperature. Temperature is a monotonic function of the average molecular kinetic energy of a substance. When a substance is heated, the kinetic energy of its molecules increases. Thus, the molecules begin vibrating/moving more and usually maintain a greater average separation. So most materials will expand as the temperature increases. The relative expansion divided by the change in temperature is called the material's coefficient of thermal expansion (CTE) and generally varies with temperature. The coefficient of thermal expansion describes how the size of an object changes with a change in temperature. Specifically, it measures the fractional change in size per degree change in temperature at a constant pressure[3,4]. When combined layer by layer after curing, the PDMS and silicone form a very strong binding force in the junction, the thermal expansion coefficient (CTE) of PDMS is smaller than silicone, which would lead to a bend of composite towards PDMS layer. The interface of two materials is well fused through SEM images. In order to make the double layer composite stretchable, the PDMS layer is cut into multiple sections. When stretching, the PDMS sections will be separate from each other. When releasing, the separated PDMS sections will retract for the resilience of silicone.

Supplementary Note 2

Mechanosensitive multiple channels of BSNG.

The multiple channels can be opened or closed by tension. To make the channels more hydrophobic for liquid flowing, some bamboo joint-liked microstructures at the bottom of the channels and reservoir are built by reversing 3D printed mold, which are shown in SEM images.

Supplementary Note 3

Contact angle test results of the silicone with and without microstructures.

The Contact angle test results (Supplementary Fig. 4) show that the deionized water has a larger contact angle with the surface of silicone with bamboo joint-liked microstructures compared to the surface of silicone without microstructures, which demonstrate that the surface of the silicone with microstructure has better hydrophobicity than the smooth surface without microstructure.

Supplementary Note 4

Specific dimensions of each part structure of BSNG.

The whole size of the BSNG is $6\ \text{cm} \times 10\ \text{cm} \times 8\ \text{mm}$. For the electrification layer of BSNG, the size of the area of bionic channels is $2\ \text{cm} \times 8\ \text{cm} \times 2\ \text{mm}$, the size of the fluid chamber is $1\ \text{cm} \times 8\ \text{cm} \times 2\ \text{mm}$. The size of the connecting pipelines between bionic channels and fluid chamber is 1 cm in length and 1.5 mm in diameter. For the induction layer of BSNG, the sizes of two electrodes are $2\ \text{cm} \times 8\ \text{cm} \times 1\ \text{mm}$ and $1\ \text{cm} \times 8\ \text{cm} \times 1\ \text{mm}$, facing to the region of bionic channels and fluid chamber of the electrification layer respectively. The thickness of the silicone layer between the electrification layer and the induction layer is 1 mm. The thickness of the silicone layer wrapped around the electrification layer and the induction layer is 2 mm.

Supplementary Note 5

Progress of Na^+ and K^+ transfer in cell membrane.

In the resting state of one electricity generation cycle of an electrocyte, the K^+

concentration is high and the Na^+ concentration is low inside the cell membrane, and opposite of them outside the cell membrane. Although Na^+ and K^+ channels are closed, the permeability of cell membranes to potassium ions far exceeds that of sodium ions. K^+ ions diffuse out of the cell membrane spontaneously and form a potential drop. When nerves are excited, the Na^+ channels switch from closed to opened and Na^+ ions flow in membrane promptly. The rapid transmembrane movement of Na^+ ions leads to an action potential (negative outside the membrane and positive inside the membrane). This is depolarization (DP) process of cell membrane. When membrane depolarization reaches a certain level leading to an action potential peak (150 mV potential difference), Na^+ channels are inactivated and closed, while K^+ channels turn to open. K^+ ions flow out promptly and the permeability of the cell membrane to sodium ions returns to a resting state, which results in a fast setback of action potential. This is repolarization (RP) process of the cell membrane[5-7].

The generation process of the transcellular potential can be concluded as follows: At rest state, both the innervated and noninnervated membrane exhibit a potential of -85 mV. When stimulated, activated acetylcholine receptors (AchRs) generate endplate potentials, triggering Na^+ channel-mediated action potentials peaking at +65 mV on the innervated membrane. The noninnervated membrane contains no voltage-gated Na+ channels and maintains resting potential at -85 mV. The result is the transcellular potential difference of an approximate value of 150 mV.

Supplementary Note 6

Outputs of the BSNG in different working modes.

The first working mode is single electrode mode. Because of the universality of contact electrification between any two different materials, the BSNG can generate electric outputs from the relative motion between the BSNG and any other materials when working as a single electrode mode. Aluminum sheet, Teflon film and human hand are used to tap the BSNG, and corresponding open circuit voltages (V_{oc}) and short-circuit transferred charges (Q_{sc}) are tested. The peak V_{oc} and the peak Q_{sc} are about 160 V and 47 nC respectively. The amplitude and polarization of V_{oc} depend on the relative

ability of losing and gaining electrons of the material contacted with the silicone. Compared with silicone, Al and human skin are tending to lose electrons and therefore are more tribo-positive, whereas PTFE is more tribo-negative. The polarizations of the V_{oc} are opposite, which are coincident with the well-established tribo-series table. The larger the difference in the ability of losing and gaining electrons between the two contacting materials, the more electrostatic charges are generated at the interface and higher output Voc can be got.

The second working mode is liquid-solid contact mode. When the BSNG is stretched along with its long axis, the polarizations of V_{oc} and Q_{sc} are same with tapping the BSNG with PTFE, but contrary to tapping BSNG with Al and hand. The results indicate that the processes of the electric charge generated by the two working modes are different.

Supplementary Note 7

Underwater output of the single-electrode mode BSNG.

The output performances of the single-electrode mode BSNG (SE-BSNG) can be largely affected by the humidity of environment. Both Voc and Isc of the SE-BSNG decrease significantly as the ambient humidity increases. In the humid environment, the surface electrostatic charges of the SE-BSNG will be screened by the adsorbed ambient water molecules. As a result, the outperformances of the SE-BSNG are attenuated severely.

Supplementary Note 8

Principle of the single-electrode mode BSNG.

When an object contacts with the BSNG, the electrification occurs at the interface between the object and the silicone, resulting in the same amount of charges with different polarities at the surface of them. However, there is no electrical potential drop between the two surfaces at this state, because the charges with opposite polarities coexist at almost the same plane. When the object is separated from the BSNG and moving away, the electrostatic charges on the surface of the silicone will induce the

ions in liquid electrodes moving to balance the electrostatic charges, forming a layer of excessive ions at the interface. At the same time, the electrical double layer formed at the interface between the metal and electrolyte (NaCl solution in this work) are polarized, forming the equal opposite excessive ions at the interface and corresponding charges in the copper wire. Electrons flow from the copper wires to the ground through the external loads till all electrostatic charges in the silicone are screened. When the object moves back to the BSNG, the electrons flow back into the interface between the copper wire and NaCl solution from the ground through the external load. Therefore, an alternative electrical signal will be generated by repeating the movements of contacting and separating between the object and the BSNG. The changing process of surface potential of the BSNG has also been simulated by software (COMSOL).

Supplementary Note 9

Characterization of the internal structure of BSNG after 50,000 times fatigue test.

After 50 thousand times uniaxial tensile test, the output performance of BSNG is not attenuated, and BSNG has a complete structure without any damage from the outside. To prove that the internal structure of BSNG also has no changes, the electrode layer on the back of the BSNG was cut open to observe whether the structure has changed. From the optical photos (Supplementary Fig. 12a and b), the internal channel structure of BSNG is intact without any damage (Red ink is injected to see the internal structure of BSNG more clearly). In addition, the internal bionic channel structure was also characterized by SEM (Supplementary Fig. 12c and d). From the SEM photos of bionic channels of BSNG, the bamboo joint-liked microstructures at the bottom of the channels is also intact. Benefiting from the small friction stress between liquid and soft matter, the bionic channels inside the BSNG is not easily damaged even after a long period of reciprocating flow of the electrification liquid.

Supplementary Note 10

Statistical analysis of motion signals in four strokes.

The data of the recorded motion signals of each swimming stroke have been further

analyzed, and the average motion amplitude and average time interval of the peaks of the motion signals of each stroke have been extracted respectively (Supplementary Figure 13). From the statistical results, we can know that the amplitudes of the leg movements are larger than the arm movements in breaststroke and backstroke, while the amplitudes of the arm movements are larger than the leg movements in the freestyle. When treading water, the arms have almost no movement and only the signals of legs' movements are caught.

The average peak intervals of the motion signals of the legs and arms in each stroke are similar, so the movement frequency of the legs and arms is almost consistent in each stroke, indicating that the arms and legs of the subjects are coordinated in each stroke. The amplitude sorting of the leg movements with four strokes from high to low is: backstroke, breaststroke, tread water, and freestyle. In contrast, the difference in the amplitudes of the arm movements is not obvious in these three strokes (except for treading water).

The time difference of each cycle is different for each stroke, indicating the frequencies of each movement in different strokes are not the same. The movement frequencies of freestyle and tread water are fast and very similar, followed by breaststroke, and backstroke is the slowest. Because in each cycle, the swimmer should complete a movement with higher speed in freestyle and tread water than that in breaststroke. The time required to complete a movement in backstroke is the longest. With the information acquired from BSNGs, we can analyze the specific case of each movement of the swimmer to estimate the physiology state under the water. For example, when the swimmer's physical strength begins to decrease, exhibiting a fatigue state with the amplitude of the motion signal decreasing and the time interval of each cycle increasing.

Supplementary Note 11

Recharging a 100 μF capacitor by BSNG after the transmitter launching a wireless signal.

The operation threshold voltage of the wireless transmitter is 2.2 V. In practical

application, it usually does not need to start charging an energy storage device from 0 V. The energy storage device should be precharged. In this case, the BSNG just need to charge a capacitor upon the operation threshold voltage for launching wireless signal. In our experiment, when a trigger signal is transmitted, the voltage of the capacitor will drop from 2.2 to about 1 V. We recharge this 100 μF capacitor from 1 V to 2.2 V within 1 hour by the BSNG in our lab.

Supplementary Note 12

Three types of applications for BSNG.

The BSNG can also be applied in other application scenarios: The first application of BSNG is as a controller. By coupling with a traditional FET, BSNG can be used for active modulation of conventional electronics, for example, to control the blinking of an LED through BSNG. Using the electrostatic potential created by triboelectrification as a "gate" voltage to tune/control electrical transport and transformation in semiconductors, BSNG has established a direct interaction mechanism between the human and electronics. In the near future, BSNG may also be used for electronic skin, human-machine interface, etc.

Another application of BSNG is on land energy harvesting. The energy harvested by a wearable BSNG can be stored and drive some portable appliances, such as healthcare monitoring, humidity sensor, temperature sensor, LCD display, etc. The third application of BSNG is acted as a wearable sensor for athletic exercise. For example, the BSNG can be used for some common movement monitoring in daily life, such as body-building and marathon.

Supplementary References

1 Lin, W., Moon, K. S. & Wong, C. P. A Combined Process of In Situ Functionalization and Microwave Treatment to Achieve Ultrasmall Thermal Expansion of Aligned Carbon Nanotube-Polymer Nanocomposites: Toward Applications as Thermal Interface Materials. *Adv Mater* **21**, 2421-2424 (2010).

2 Pye, J. E. & Roth, C. B. Physical Aging of Polymer Films Quenched and Measured Free-Standing via Ellipsometry: Controlling Stress Imparted by Thermal Expansion Mismatch between Film and Support. *Macromolecules* **46**, 9455-9463 (2013).

3 Tipler, P. A. & Mosca, G. *Physics for scientists and engineers*. Macmillan (2007).

4 Bönisch, M. *et al.* Giant thermal expansion and α-precipitation pathways in Ti-alloys. *Nat Commun* **8**, 1429 (2017).

5 Stuhmer, W. *et al.* Structural Parts Involved in Activation and Inactivation of the Sodium-Channel. *Nature* **339**, 597-603 (1989).

6 Gadsby, D. C., Rakowski, R. F. & Deweer, P. Extracellular Access to the Na,K Pump - Pathway Similar to Ion Channel. *Science* **260**, 100-103 (1993).

7 Catterall, W. A. From ionic currents to molecular mechanisms: The structure and function of voltage-gated sodium channels. *Neuron* **26**, 13-25 (2000).
